# *catena*-Poly[[[triaqua­cadmium(II)]-μ-2,2′-[oxalylbis(azanediyl)]diacetato-κ^2^ *O*,*O*′] dihydrate]

**DOI:** 10.1107/S1600536809052015

**Published:** 2009-12-09

**Authors:** Fangxia Zhou

**Affiliations:** a Department of Chemistry , Jining University, Shandong 273155, People’s Republic of China

## Abstract

The structure of the polymeric title complex, {[Cd(C_6_H_6_N_2_O_6_)(H_2_O)_3_]·2H_2_O}_*n*_, consists of chains running parallel to [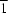
01] in which the oxamidato ligand, deprotonated only at the carboxyl­ate groups, acts as a bridging bis-monodentate ligand. The Cd atom and the O atom of a coordinated water mol­ecule are located on a twofold axis. The coordination geometry around the Cd atom is distorted trigonal-pyramidal. In the crystal structure, neighbouring chains are linked into a three-dimensional network by inter­chain O—H⋯O and N—H⋯O hydrogen bonds.

## Related literature

For the crystal structure of the corresponding copper(II) compound, see: Lloret *et al.* (1992 ▶).
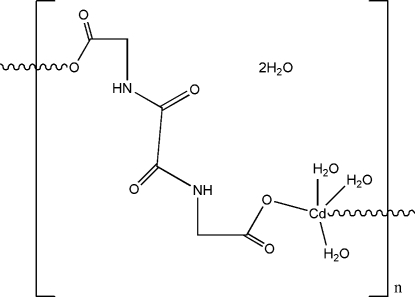


## Experimental

### 

#### Crystal data

[Cd(C_6_H_6_N_2_O_6_)(H_2_O)_3_]·2H_2_O
*M* *_r_* = 404.61
Monoclinic, 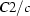

*a* = 7.0898 (14) Å
*b* = 8.0306 (16) Å
*c* = 23.396 (5) Å
β = 92.06 (3)°
*V* = 1331.2 (5) Å^3^
*Z* = 4
Mo *K*α radiation
μ = 1.70 mm^−1^
*T* = 293 K
0.23 × 0.18 × 0.15 mm

#### Data collection

Bruker SMART CCD diffractometer
Absorption correction: multi-scan (*SADABS*; Sheldrick, 1996 ▶) *T*_min_ = 0.696, *T*_max_ = 0.785
3445 measured reflections
1214 independent reflections
1171 reflections with *I* > 2σ(*I*)
*R*_int_ = 0.038

#### Refinement

*R*[*F* ^2^ > 2σ(*F* ^2^)] = 0.023
*wR*(*F* ^2^) = 0.058
*S* = 1.07
1214 reflections
93 parameters
H-atom parameters constrained
Δρ_max_ = 0.50 e Å^−3^
Δρ_min_ = −0.53 e Å^−3^

### 

Data collection: *SMART* (Bruker, 1998 ▶); cell refinement: *SAINT* (Bruker, 1998 ▶); data reduction: *SAINT*; program(s) used to solve structure: *SHELXS97* (Sheldrick, 2008 ▶); program(s) used to refine structure: *SHELXL97* (Sheldrick, 2008 ▶); molecular graphics: *SHELXTL* (Sheldrick, 2008 ▶); software used to prepare material for publication: *SHELXL97* and *PLATON* (Spek, 2009 ▶).

## Supplementary Material

Crystal structure: contains datablocks I, global. DOI: 10.1107/S1600536809052015/rz2395sup1.cif

Structure factors: contains datablocks I. DOI: 10.1107/S1600536809052015/rz2395Isup2.hkl

Additional supplementary materials:  crystallographic information; 3D view; checkCIF report

## Figures and Tables

**Table 1:** Hydrogen-bond geometry (Å, °)

*D*—H⋯*A*	*D*—H	H⋯*A*	*D*⋯*A*	*D*—H⋯*A*
N1—H1⋯O6^i^	0.86	2.31	3.024 (3)	141
O4—H4*W*1⋯O1^ii^	0.88	1.78	2.654 (2)	171
O6—H6*W*2⋯O2^iii^	0.93	2.03	2.869 (3)	150
O5—H5*W*⋯O4^iv^	0.83	1.89	2.717 (3)	170
O4—H4*W*2⋯O6	0.91	1.83	2.733 (3)	170
O6—H6*W*1⋯O3	0.88	1.97	2.839 (3)	170

Symmetry codes: (i) 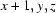; (ii) 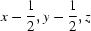; (iii) 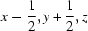; (iv) 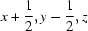.

**Fig. 1.**
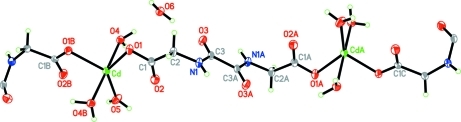
The polymeric structure of the title compound, with displacement ellipsoids drawn at the 30% probability level. Lattice water molecules generated by symmetry are omitted. [Symmetry codes: (A) 3/2-x, 1/2-y, -z; (B) 1-x, y, 1/2-z; (C) 1/2+x, 1/2-y, -1/2+z].

**Fig. 2.**
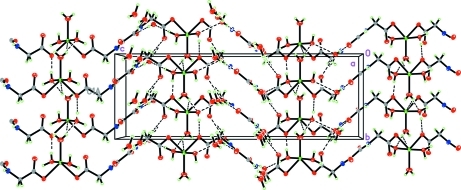
Packing diagram of the title compound viewed along the a axis. Interchain H bonds are shown as dashed lines.

## supplementary crystallographic information

### Comment

N-Substituted and N,N'-disubstituted oxamides have played an important role in the design of new polymetallic systems. The versatility of these ligands is based on the wide variety of substituted derivatives which can be synthesized, yielding different numbers of chelate rings with different donor atoms, and on their easy *cis-trans* conformational change affording symmetric and asymmetric oxamidato bridges. A new polymeric cadmium(II) complex bridged by a symmetrical oxamide-N,N'-diacetic acid ligand has been synthesized and its crystal structure is reported herein.

The title compound (Fig. 1) is a polymeric cadmium(II) complex forming one-dimensional chains parallel to [1 0 1]. The Cd and the oxygen atom of a coordinated water molecules are located on a two-fold axis and the midpoint of the oxamide C—C bond on an inversion centre. The ligand is deprotonated  only at the terminal carboxylate groups and acts as a bis-monodentate bridge. The coordination geometry around the Cd atom is distorted trigonal pyramidal, with atoms O5, O1 and O1^i^ [symmetry code: (i) 1-x, y, 1/2-z] at the equatorial plane and atoms O4 and O4^i^ at the apical positions [O4—Co1—O4^i^ = 178.29 (8)°]. The sum of the O—Cd—O angles within the equatorial plane is 359.99 (9)°. The structure is similar to that previously reported for the copper(II) complex (Lloret *et al.*, 1992). The cadmium-cadmium separation within the chain 12.369 (4) Å. Strong interchain N—H···O and O—H···O hydrogen bonds (Table 1) result in the formation of a three-dimensional network (Fig. 2).

### Experimental

To a stirred methanol solution (10 ml) containing Cd(NO_3_)_2_^.^3H_2_O (0.0581 g, 0.2 mmol) was added dropwise a methanol solution (10 ml) of oxamide-N,N'-diacetic acid (0.0408 g, 0.2 mmol) and piperidine. The mixture was stirred quickly at 323 K for 5 h. The resulting solution at pH = 3 was filtered and the filtrate was kept at room temperature. Green crystals suitable for X-ray analysis were obtained from the filtrate by slow evaporation for 3 days (yield: 65%) Analysis, calculated for C_6_H_16_N_2_O_11_Cd: C 17.81, H 3.99; N 6.92%; found: C 17.89, H 3.97, N, 6.96%.

### Refinement

Water H atoms were located in a difference Fourier map and isotropically refined with *U*_iso_(H) = 0.08 Å^2^. All other H atoms were positioned geometrically and constrained to ride on their parent atoms, with C—H = 0.97 Å, N—H = 0.86 Å, and with *U*_iso_(H) = 1.2*U*_eq_(C, N).

### Crystal data

**Table:**

[Cd(C_6_H_6_N_2_O_6_)(H_2_O)_3_]·2H_2_O	*F*(000) = 808
*M**_r_* = 404.61	*D*_x_ = 2.019 Mg m^−^^3^
Monoclinic, *C*2/*c*	Mo *K*α radiation, λ = 0.71073 Å
Hall symbol: -C 2yc	Cell parameters from 3445 reflections
*a* = 7.0898 (14) Å	θ = 3.5–25.2°
*b* = 8.0306 (16) Å	µ = 1.70 mm^−^^1^
*c* = 23.396 (5) Å	*T* = 293 K
β = 92.06 (3)°	Block, green
*V* = 1331.2 (5) Å^3^	0.23 × 0.18 × 0.15 mm
*Z* = 4	

### Data collection

**Table:**

Bruker SMART CCD diffractometer	1214 independent reflections
Radiation source: fine-focus sealed tube	1171 reflections with *I* > 2σ(*I*)
graphite	*R*_int_ = 0.038
φ and ω scans	θ_max_ = 25.2°, θ_min_ = 3.5°
Absorption correction: multi-scan (*SADABS*; Sheldrick, 1996)	*h* = −7→8
*T*_min_ = 0.696, *T*_max_ = 0.785	*k* = −9→9
3445 measured reflections	*l* = −25→28

### Refinement

**Table:**

Refinement on *F*^2^	Secondary atom site location: difference Fourier map
Least-squares matrix: full	Hydrogen site location: inferred from neighbouring sites
*R*[*F*^2^ > 2σ(*F*^2^)] = 0.023	H-atom parameters constrained
*wR*(*F*^2^) = 0.058	*w* = 1/[σ^2^(*F*_o_^2^) + (0.032*P*)^2^ + 1.2127*P*] where *P* = (*F*_o_^2^ + 2*F*_c_^2^)/3
*S* = 1.07	(Δ/σ)_max_ < 0.001
1214 reflections	Δρ_max_ = 0.50 e Å^−^^3^
93 parameters	Δρ_min_ = −0.53 e Å^−^^3^
0 restraints	Extinction correction: *SHELXL*, Fc^*^=kFc[1+0.001xFc^2^λ^3^/sin(2θ)]^-1/4^
Primary atom site location: structure-invariant direct methods	Extinction coefficient: 0.0057 (5)

### Special details

**Table:**

Geometry. All esds (except the esd in the dihedral angle between two l.s. planes) are estimated using the full covariance matrix. The cell esds are taken into account individually in the estimation of esds in distances, angles and torsion angles; correlations between esds in cell parameters are only used when they are defined by crystal symmetry. An approximate (isotropic) treatment of cell esds is used for estimating esds involving l.s. planes.
Refinement. Refinement of F^2^ against ALL reflections. The weighted R-factor wR and goodness of fit S are based on F^2^, conventional R-factors R are based on F, with F set to zero for negative F^2^. The threshold expression of F^2^ > 2sigma(F^2^) is used only for calculating R-factors(gt) etc. and is not relevant to the choice of reflections for refinement. R-factors based on F^2^ are statistically about twice as large as those based on F, and R- factors based on ALL data will be even larger.

### Fractional atomic coordinates and isotropic or equivalent isotropic displacement parameters (Å^2^)

**Table:**

	*x*	*y*	*z*	*U*_iso_*/*U*_eq_	
C1	0.6801 (3)	0.3969 (3)	0.15103 (10)	0.0264 (5)	
C2	0.7678 (4)	0.4894 (3)	0.10180 (11)	0.0300 (6)	
H1A	0.6764	0.5681	0.0859	0.036*	
H1B	0.8756	0.5525	0.1166	0.036*	
C3	0.7039 (4)	0.3045 (3)	0.02244 (11)	0.0272 (6)	
N1	0.8284 (3)	0.3821 (3)	0.05672 (9)	0.0308 (5)	
H1	0.9470	0.3680	0.0519	0.037*	
O1	0.5996 (2)	0.4896 (2)	0.18701 (8)	0.0328 (4)	
O2	0.6869 (3)	0.2444 (3)	0.15559 (9)	0.0407 (5)	
O3	0.5325 (3)	0.3144 (2)	0.02483 (9)	0.0377 (5)	
O4	0.2191 (3)	0.3021 (2)	0.19554 (8)	0.0304 (4)	
H4W1	0.1704	0.2020	0.1897	0.080*	
H4W2	0.2397	0.3493	0.1608	0.080*	
O5	0.5000	0.0250 (3)	0.2500	0.0437 (7)	
H5W	0.5695	−0.0341	0.2305	0.080*	
O6	0.2377 (2)	0.4466 (2)	0.09015 (8)	0.0362 (4)	
H6W1	0.3381	0.4129	0.0726	0.080*	
H6W2	0.2583	0.5541	0.1036	0.080*	
Cd	0.5000	0.29781 (3)	0.2500	0.02762 (15)	

### Atomic displacement parameters (Å^2^)

**Table:**

	*U*^11^	*U*^22^	*U*^33^	*U*^12^	*U*^13^	*U*^23^
C1	0.0266 (12)	0.0301 (13)	0.0225 (12)	−0.0053 (10)	−0.0004 (9)	−0.0037 (11)
C2	0.0376 (14)	0.0280 (13)	0.0250 (13)	−0.0056 (11)	0.0073 (11)	−0.0049 (11)
C3	0.0346 (14)	0.0277 (14)	0.0198 (13)	0.0017 (10)	0.0074 (11)	0.0004 (10)
N1	0.0326 (11)	0.0351 (12)	0.0253 (11)	−0.0017 (9)	0.0091 (9)	−0.0060 (10)
O1	0.0391 (10)	0.0324 (10)	0.0277 (10)	−0.0058 (8)	0.0108 (8)	−0.0048 (8)
O2	0.0580 (13)	0.0273 (10)	0.0375 (12)	0.0003 (10)	0.0107 (10)	0.0027 (9)
O3	0.0310 (11)	0.0460 (12)	0.0364 (11)	0.0035 (8)	0.0058 (9)	−0.0099 (9)
O4	0.0337 (10)	0.0260 (10)	0.0316 (11)	−0.0015 (7)	0.0027 (8)	−0.0023 (7)
O5	0.0417 (15)	0.0282 (14)	0.063 (2)	0.000	0.0247 (14)	0.000
O6	0.0353 (10)	0.0368 (11)	0.0368 (11)	0.0000 (8)	0.0055 (8)	−0.0020 (8)
Cd	0.0324 (2)	0.02517 (19)	0.02557 (19)	0.000	0.00427 (11)	0.000

### Geometric parameters (Å, °)

**Table:**

C1—O2	1.230 (3)	O1—Cd	2.2621 (18)
C1—O1	1.274 (3)	O4—Cd	2.326 (2)
C1—C2	1.522 (3)	O4—H4W1	0.8839
C2—N1	1.440 (3)	O4—H4W2	0.9134
C2—H1A	0.9700	O5—Cd	2.191 (3)
C2—H1B	0.9700	O5—H5W	0.8320
C3—O3	1.221 (3)	O6—H6W1	0.8775
C3—O3	1.221 (3)	O6—H6W2	0.9280
C3—N1	1.326 (4)	Cd—O1^ii^	2.2621 (18)
C3—C3^i^	1.531 (5)	Cd—O4^ii^	2.326 (2)
N1—H1	0.8600		
			
O2—C1—O1	122.8 (2)	C1—O1—Cd	100.97 (15)
O2—C1—C2	122.4 (2)	Cd—O4—H4W1	113.2
O1—C1—C2	114.8 (2)	Cd—O4—H4W2	109.4
N1—C2—C1	113.7 (2)	H4W1—O4—H4W2	108.3
N1—C2—H1A	108.8	Cd—O5—H5W	124.8
C1—C2—H1A	108.8	H6W1—O6—H6W2	109.0
N1—C2—H1B	108.8	O5—Cd—O1^ii^	132.90 (4)
C1—C2—H1B	108.8	O5—Cd—O1	132.90 (4)
H1A—C2—H1B	107.7	O1^ii^—Cd—O1	94.19 (9)
O3—C3—N1	125.7 (2)	O5—Cd—O4	90.86 (4)
O3—C3—N1	125.7 (2)	O1^ii^—Cd—O4	93.76 (7)
O3—C3—C3^i^	121.3 (3)	O1—Cd—O4	85.08 (7)
O3—C3—C3^i^	121.3 (3)	O5—Cd—O4^ii^	90.86 (4)
N1—C3—C3^i^	113.1 (3)	O1^ii^—Cd—O4^ii^	85.08 (7)
C3—N1—C2	121.0 (2)	O1—Cd—O4^ii^	93.76 (7)
C3—N1—H1	119.5	O4—Cd—O4^ii^	178.29 (8)
C2—N1—H1	119.5		

Symmetry codes:  (i) −*x*+3/2, −*y*+1/2, −*z*; (ii) −*x*+1, *y*, −*z*+1/2.

### Hydrogen-bond geometry (Å, °)

**Table:**

*D*—H···*A*	*D*—H	H···*A*	*D*···*A*	*D*—H···*A*
N1—H1···O6^iii^	0.86	2.31	3.024 (3)	141
O4—H4W1···O1^iv^	0.88	1.78	2.654 (2)	171
O6—H6W2···O2^v^	0.93	2.03	2.869 (3)	150
O5—H5W···O4^vi^	0.83	1.89	2.717 (3)	170
O4—H4W2···O6	0.91	1.83	2.733 (3)	170
O6—H6W1···O3	0.88	1.97	2.839 (3)	170

Symmetry codes: (iii) *x*+1, *y*, *z*; (iv) *x*−1/2, *y*−1/2, *z*; (v) *x*−1/2, *y*+1/2, *z*; (vi) *x*+1/2, *y*−1/2, *z*.
